# Pemphigus herpetiformis: A case report of response to dupilumab after failure of multiple conventional therapies

**DOI:** 10.1016/j.jdcr.2025.10.022

**Published:** 2025-11-14

**Authors:** Fabiola Pabón-González, Iancarlos Jiménez-Sacarello, José Cuesta-Camuñas, Oscar Nevarez-Pomales, Luis G. García-Guzmán, Melanie Medina-Figueroa

**Affiliations:** a Department of Dermatology, University of Puerto Rico School of Medicine, San Juan, Puerto Rico; b School of Medicine, University of Puerto Rico, Medical Sciences Campus, San Juan, Puerto Rico

**Keywords:** dupilumab, pemphigus herpetiformis

## Introduction

Pemphigus herpetiformis (PH) is a rare variant of pemphigus diseases, occurring in <10% of patients with pemphigus and with no tendency toward a specific sex, age group, or ethnicity.^1^ It is classified distinctly from pemphigus because of its clinical presentation and benign disease course.^2^ Like other autoimmune bullous disease, PH commonly presents with vesicles, bullae, or papules surrounded by erythema and with severe pruritus.^1^ PH lesions tend to be annular in shape, commonly appearing in the trunk and proximal extremities with sparing of mucous membranes.^1^^,^^2^ The exact mechanism by which autoantibodies produce the characteristic skin lesions seen in PH is still not known. Histologically, it appears with spongiosis, eosinophilic infiltrate, and an intraepidermal split without acantholysis.^2^ There are no specific guidelines for PH management, but treatments include systemic corticosteroids, immunosuppressants, dapsone, intravenous immunoglobulins, and plasmapheresis.

## Case report

**Fig 1:**
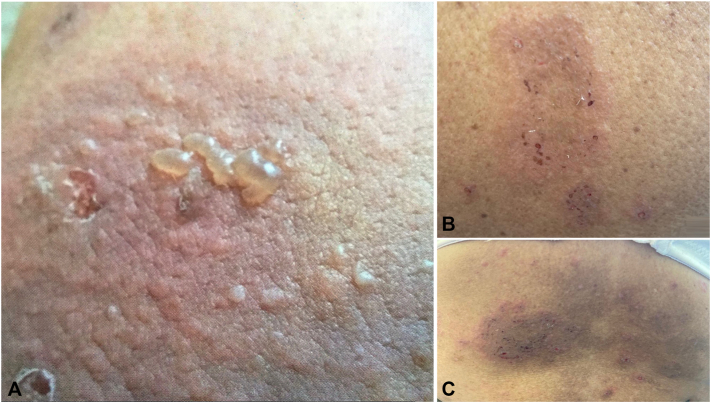
Clinical presentation of: **(A)** annular erythematous plaque with grouped vesicles and some erosions on left lower extremity, **(B)** annular erythematous with multiple excoriated papules on upper back, and **(C)** multiple scattered erythematous papules some coalescing to a large plaque with associated postinflammatory hyperpigmentation on lower back.

**Fig 2:**
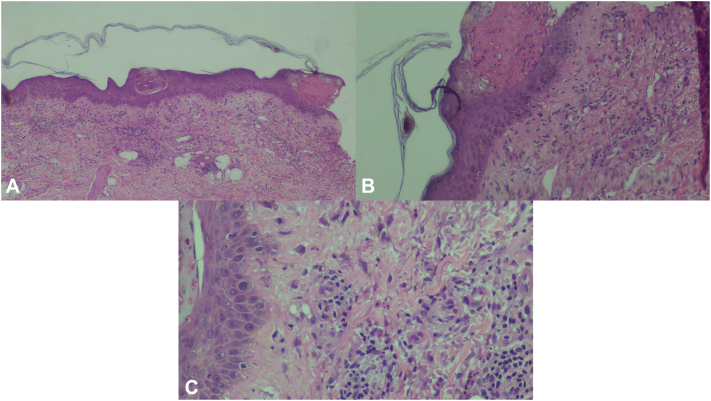
Histologic findings on punch biopsy **(A)** epidermis showing spongiotic vesiculopustules and abundant eosonophils. (**B)** Intraepidermal vesicles with focal necrosis. (**C)** Mixed inflammatory infiltrate with eosinophils.

A 72-year-old female patient from Puerto Rico, with a long history of PH, has experienced recurrent bouts of disease associated with significant eosinophilia when active. Recurrent lesions present as multiple annular and erythematous papules and vesicles on her back (Fig 1) and arms. At the time of diagnosis, histopathologic examination of a skin biopsy showed spongiotic dermatitis with spongiotic vesiculopustules and abundant eosinophils (Fig 2). Direct immunofluorescence was positive, showing intraepidermal intercellular staining of IgG and C3. Serum studies were negative for DSG1, DSG3, BPAG180, and BP230 antibodies.

Previous treatment with dapsone resulted in the resolution of lesions but was discontinued due to elevated creatinine. Lesions appeared again, and the patient was started on rituximab, with improvement of lesions and relief of pruritus, but recurring months after. Given treatment failures with more established therapies and the persistent eosinophilia in this patient, the decision was made to attempt treatment with dupilumab. The patient was started on a 600 mg loading dose followed by 300 mg every other week. Within 3 months of starting therapy, the patient had complete resolution of lesions. She continues to have total control of the disease at the 10-month follow-up.

## Discussion

PH is an uncommon clinical variant of pemphigus that constitutes a small percentage of pemphigus cases. PH clinically presents as pruritic, annular, or herpetiform lesions that are similar to those of dermatitis herpetiformis and other subepidermal blistering diseases.^1^ PH usually differs on histology from other types of pemphigus due to spongiosis and epidermal microabscesses, and an inflammatory infiltrate with eosinophilic predominance.^1^^,^^2^ Direct immunofluorescence can show intraepidermal blistering and intercellular IgG and C3 deposition. The treatment of PH generally begins with dapsone as first choice and is followed by the use of systemic corticosteroids and/or immunosuppressants such as mycophenolate mofetil, if necessary.^2^^,^^3^

Rituximab, a monoclonal anti-CD20 antibody, has become a first-line biologic treatment for pemphigus vulgaris and foliaceus and has been shown to have efficacy in PH as well.^4^^,^^5^ In this patient, however, the PH recurred after initial improvement with rituximab, demonstrating the heterogeneity of response to treatment of the pemphigus subtypes.

To our knowledge, this is the first reported case of PH successfully treated with dupilumab reported in the literature. Clinical remission was induced in this patient on dupilumab, a monoclonal antibody targeting the interleukin (IL)-4 receptor α subunit that blocks IL-4 and IL-13 signaling.^6^ Dupilumab is mainly approved for atopic dermatitis and other T helper type 2-mediated diseases but has recently proven to be effective in some bullous skin disorders.^7^ A previous study showed that increased IL-4 and IL-10 are involved in T cells induction and autoimmune phenomena of pemphigus vulgaris and pemphigus foliaceous.^8^ Recent reports indicate response to dupilumab in 2 cases of pemphigus vulgaris and foliaceous with disease control lasting more than a year.^9^ Interestingly, PH has been said to be mainly Th2-mediated. Proinflammatory cytokines such as IL-4 have been suggested to play a role in the IL-31 pathway and increased epidermal expression of IL-31 receptor A.^10^ Of note, our patient’s history revealed multiple episodes of eosinophilia associated with disease activity, which improved with prednisone courses but recurred upon discontinuation. A previous study of 158 patients with PH reported eosinophilia in 15% of cases.^2^ Because of the parallels with its mechanism in other bullous disorders, we selected dupilumab to specifically target the Th2-mediated eosinophil chemotaxis and activation implicated in the patient’s disease.^6^ Dupilumab, by inhibition of IL-4 inflammatory effects and regulating the Th2 immune pathway, might have a role in the treatment of PH, as seen in our case.

This case highlights the importance of investigating other immunomodulatory treatments in patients with PH who are resistant to standard drugs, including rituximab. It also suggests that dupilumab, through its inhibition of inflammatory cytokines and Th2 cell induction in pemphigus pathogenesis, can be an effective adjunct or alternative treatment in certain cases. Further research is needed to determine the efficacy and safety of dupilumab in PH and similar disorders.

## Conflicts of interest

None disclosed.

## References

[1] Kasperkiewicz M., Kowalewski C., Jabłońska S. (2014). Pemphigus herpetiformis: from first description until now. J Am Acad Dermatol.

[2] Costa L.M.C., Cappel M.A., Keeling J.H. (2019). Clinical, pathologic, and immunologic features of pemphigus herpetiformis: a literature review and proposed diagnostic criteria. Int J Dermatol.

[3] Sánchez-Pérez A.P., Urbina-Calderón F., Pretell-Vera J. (2021). A case of pemphigus herpetiformis with excellent response to mycophenolate mofetil. Acta Dermatovenerol Alp Pannonica Adriat.

[4] Marniquet M.E., Joly P., Dansette D., Fenot M. (2022). A case of pemphigus herpetiformis successfully treated with rituximab. J Eur Acad Dermatol Venereol.

[5] Hamed M., Krausz J., Ziv M., Barak E.C. (2024). Pediatric pemphigus herpetiformis treated with rituximab. JAAD Case Rep.

[6] Olbrich H., Sadik C.D., Ludwig R.J., Thaçi D., Boch K. (2023). Dupilumab in inflammatory skin diseases: a systematic review. Biomolecules.

[7] Cao P., Xu W., Zhang L. (2022). Rituximab, omalizumab, and dupilumab treatment outcomes in bullous pemphigoid: a systematic review. Front Immunol.

[8] Satyam A., Khandpur S., Sharma V.K., Sharma A. (2009). Involvement of T(H)1/T(H)2 cytokines in the pathogenesis of autoimmune skin disease-pemphigus vulgaris. Immunol Invest.

[9] Jiang C., Adjei S., Santiago S., Lu J., Duran M., Tyring S. (2023). Novel use of dupilumab in pemphigus vulgaris and pemphigus foliaceus. JAAD Case Rep.

[10] Morais K.L., Miyamoto D., Orfali R.L. (2020). Increased expression of in situ IL-31RA and circulating CXCL8 and CCL2 in pemphigus herpetiformis suggests participation of the IL-31 family in the pathogenesis of the disease. J Eur Acad Dermatol Venereol.

